# Commentary: Genome-wide association study identifies 74 loci associated with educational attainment

**DOI:** 10.3389/fnmol.2017.00023

**Published:** 2017-01-31

**Authors:** Félix Hernández, Jesús Ávila

**Affiliations:** ^1^Centro de Biología Molecular Severo Ochoa (CSIC-UAM)Madrid, Spain; ^2^Centro de Investigación Biomédica en Red Sobre Enfermedades Neurodegenerativas (CIBERNED, ISCIII)Madrid, Spain

**Keywords:** Alzheimer's disease, educational attainment, SNP, tau, tauopathies

It is generally assumed that social and other environmental factors like poverty are involved in educational attainment. However, a recently published genome-wide association study (GWAS) for educational attainment of 293,723 individuals (Okbay et al., 2016) identified 74 loci associated with the number of years of schooling completed, with eight of them showing the highest association. One of these genes showing a single-nucleotide polymorphism (SNP) linked to educational attainment was located on chromosome 17. The SNP was rs192818565 (dbSNP ID: has merged into rs62056842 T/G) in position chr17:45914149 (according to GRCh38.p7 assembly of human genome), located within the first intron on the MAPT gene (microtubule associated protein tau, Figure 1A; http://www.ncbi.nlm.nih.gov/projects/SNP/snp_ref.cgi?rs=62056842).

**Figure 1:**
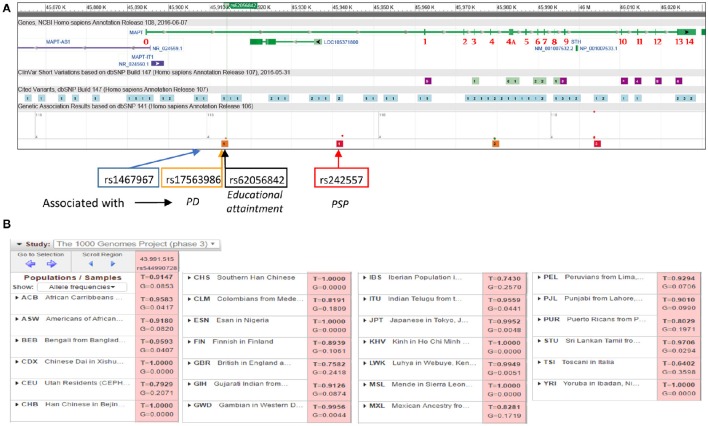
**MAPT gene**. A single tau gene located on the human chromosome 17 is transcribed into the corresponding nuclear RNA that, by alternative splicing, yields several tau mRNAs. **(A)** Exons are labeled in red numbers. *Sth* indicates the existence of a DNA sequence encoding the protein saithoin within the intron between exons 9 and 10. ncRNAs MAPT-AS1, MAPT-IT1 and likely LOC105371800 are shown. Light blue boxes: SNP present in MAPT gene; purple boxes: SNP labeled as “probable pathogenic” or “pathogenic”; red and orange boxes: SNPs that genetic association studies have linked to a strong risk to major diseases (Parkinson disease –rs17563986- and progressive supranuclear palsy –rs242557-). Some SNPs used to describe MAPT haplotypes are flanking the SNP here commented on (rs62056842) and are located within that first intron (rs1467967 and rs242557). **(B)** Allele frequencies of SNP rs192818565 in diverse human populations (rs544990728 in 1000 genomes data base). Data and images have been taken from https://www.ncbi.nlm.nih.gov/variation/tools/1000genomes/.

The human tau gene is located on chromosome 17: 45,894,382–46,028,334, and it contains 16 exons that by historical reasons have been numbered as shown in Figure 1A. Tau is mainly expressed in neurons, and by alternative splicing of exons 2, 3, and 10 yields different mRNA species in the central nervous system (Andreadis, 2005). Alternative splicing of exon 10 produces tau isoforms with either three (3R-tau, lacking exon 10) or four (4R–tau, including exon 10) tubulin/microtubule binding repeats. The locus where MAPT gene is present has been divided into two haplotypes, H1 and H2. Haplotype H2 is inverted with respect to H1. The inversion encompasses a number of genes (CRHR1, IMP5, MAPT, and NSF) and the absence of recombination between them has resulted in both haplotypes defined among other marks by 8 SNP (for a review see Caffrey and Wade-Martins, 2007). Non-inverted H1 haplotypes are more susceptibility to tauopathies and its promoter is more efficient at transcription level compared with H2 promoter (Kwok et al., 2004).

Tau protein is altered in many diseases, from Alzheimer's disease (AD) to Parkinson's disease (PD) and, as has been recently described, Huntington's disease (Avila et al., 2004; Fernández-Nogales et al., 2014; Iqbal et al., 2016). Tau missense mutations have been described in FTDP-17 while in other tauopathies tau levels or the 3R/4R-tau ratio are altered. While SNPs are roughly homogeneously distributed throughout the gene (Figure 1A, light blue boxes), clinical association studies have shown that SNPs labeled as “probably pathogenic” or “pathogenic” are mainly located at the 3′-end of the gene, where the coding exons are located (Figure 1A, purple boxes, http://www.ncbi.nlm.nih.gov/projects/SNP/). Conversely, the first intron where the SNP linked to educational attainment was located, has few of those. Interestingly, genetic association studies have found associations between SNPs in the first intron (close to rs62056842; Figure 1A, red and orange boxes) and a strong associated risk to major diseases. Furthermore, two SNPs used to describe MAPT haplotypes are flanking the above mentioned SNP and are located within that first intron (rs1467967-position chr17:45908813- and rs242557 –position chr17:45942346-) (Myers et al., 2007).

Few studies have focused on intron 0. As in many eukaryotic genes (Bradnam and Korf, 2008), the first intron is the longest one found in the MAPT gene likely because it harbors *cis* regulatory sequences (Chorev and Carmel, 2012). MAPT promoter presents two main characteristic: absence of TATA and CAAT boxes and a big G+C content (Gao et al., 2005). Thus, methylation of CpG islands present in exon and intron 0 can modulate tau expression likely altering accessibility to binding factors and transcription initiation factors (Caillet-Boudin et al., 2015). With these ideas in mind, it is tempting to speculate that rs62056842 SNP might affect that regulatory system. In fact CpG island close to rs242557 SNP is hypomethylated in PSP (Huin et al., 2016) and H1 haplotype increases risk for tauopathy via differential methylation (Li et al., 2014).

In addition, taking into account that this first intron harbors several ncRNA it can be hypothesized that the above mentioned SNP could alter some of them. MAPT-AS1 is an 840 bp long ncRNA transcribed from the anti-sense strand of the MAPT which has an inhibitory effect on MAPT promoter activity (Coupland et al., 2016). Interestingly, a significant decrease in MAPT-AS1 expression has been observed in PD (Coupland et al., 2016). Thus, it might be suggested that considering that the rs62056842 is close to the MAPT-AS1 promoter, its expression can be modulated by that particular SNP.

The study here commented on by Okbay et al. has only examined people of European ancestry and it is unclear whether the observed results apply to those with roots in other regions (Figure 1B). In fact, the 1000 genomes project (www.ncbi.nlm.nih.gov/variation/tools/1000genomes/) shows that, although rs62056842 presents an allele frequency of *T* = 0.9147 and *G* = 0.0853, being the allele *T* the one associated with higher values of educational attainment in the meta-analysis carried out by the authors, that frequency shows great variation and, for example, it is not present in people from Africa or China where *T* = 1. Overall, this study shows the importance of tau protein not only in learning, as different animal models have demonstrated, but also in educational attainment, being one of the genetic factors involved.

## Author contributions

All authors listed, have made substantial, direct and intellectual contribution to the work, and approved it for publication.

### Conflict of interest statement

The authors declare that the research was conducted in the absence of any commercial or financial relationships that could be construed as a potential conflict of interest.
